# Multiple Chronic Conditions Before Pregnancy and Risk of Adverse Maternal Health Outcomes: Population‐Based Cohort Study

**DOI:** 10.1111/1471-0528.18347

**Published:** 2025-09-03

**Authors:** Hilary K. Brown, Kinwah Fung, Eyal Cohen, Cindy‐Lee Dennis, Sonia M. Grandi, Laura C. Rosella, Catherine Varner, Simone N. Vigod, Walter P. Wodchis, Joel G. Ray

**Affiliations:** ^1^ Department of Health and Society University of Toronto Scarborough Toronto Ontario Canada; ^2^ ICES Toronto Ontario Canada; ^3^ Hospital for Sick Children Toronto Ontario Canada; ^4^ Lawrence Bloomberg Faculty of Nursing, University of Toronto Toronto Ontario Canada; ^5^ Lunenfeld‐Tannenbaum Research Institute, Mount Sinai Hospital Toronto Ontario Canada; ^6^ Dalla Lana School of Public Health, University of Toronto Toronto Ontario Canada; ^7^ Mount Sinai Hospital Toronto Ontario Canada; ^8^ Department of Family and Community Medicine Temerty Faculty of Medicine, University of Toronto Toronto Ontario Canada; ^9^ Women's College Research Institute, Women's College Hospital Toronto Ontario Canada; ^10^ Institute for Health Policy Management and Evaluation, University of Toronto Toronto Ontario Canada; ^11^ Li Ka Shing Knowledge Institute Unity Health Toronto Toronto Ontario Canada

**Keywords:** cohort studies, maternal mortality, multimorbidity, pregnancy complications

## Abstract

**Objective:**

To assess the risks of perinatal emergency department (ED) use, hospitalisation and severe maternal morbidity or mortality (SMM‐M) associated with preconception MCC, according to the number of chronic conditions, complex MCC and co‐occurring cardiometabolic conditions.

**Design:**

Population‐based cohort study.

**Setting:**

Ontario, Canada.

**Population:**

Females aged 13–54 years, with a recognised pregnancy, 2012–2021.

**Methods:**

Modified Poisson regression was used to generate adjusted relative risks (aRRs) according to the number of chronic conditions, complex MCC (≥ 3 chronic conditions affecting ≥ 3 body systems) and co‐occurring cardiometabolic conditions. aRRs were adjusted for age, parity, income quintile, rurality and immigrant/refugee status.

**Main Outcome Measures:**

ED use, hospitalisation and SMM‐M from the estimated date of conception to 42 days postpartum.

**Results:**

In total, 894 042 individuals had no pre‐pregnancy chronic condition; 357 398 had 1; 94 427 had 2; and 27 326 had ≥ 3 chronic conditions. Relative to those without a chronic condition, the aRR for ED use increased with 1 (1.26, 95% CI 1.25–1.27), 2 (1.55, 1.54–1.56) and ≥ 3 (1.86, 1.85–1.88) conditions. For hospitalisations, the corresponding aRRs were 1.45 (1.43–1.47), 2.06 (2.02–2.10) and 3.18 (3.09–3.27). For SMM‐M, the corresponding aRRs were 1.38 (1.35–1.42), 1.82 (1.75–1.90) and 2.75 (2.59–2.92). SMM‐M risk was even more pronounced with complex MCC (aRR 2.92, 95% CI 2.72–3.14), and ≥ 3 cardiometabolic conditions (aRR 5.45, 95% CI 4.29–6.91).

**Conclusions:**

MCC, especially complex or cardiometabolic MCC, is associated with elevated risk of maternal morbidity. Multidisciplinary patient‐centred care may mitigate these risks.

## 1 Introduction

Most healthcare is organised around the management of single and acute conditions. However, more than half of adults globally have a chronic condition such as diabetes mellitus or major depression [1], and one in three adults have ‘multiple chronic conditions’ (MCC) [2]. MCC has significant societal cost as it is associated with disproportionate healthcare use, adverse health outcomes and premature mortality [3, 4, 5]. While initially considered an issue of the ageing population [6], MCC is now increasingly seen in young and middle‐aged adults because of rising rates of childhood obesity, poor nutrition, physical inactivity and other chronic disease risk factors [2]. Recent studies suggest that as many as one in six pregnant women have MCC [7, 8, 9], showing the need for better data on maternal health outcomes in this ever growing group.

Studies suggest a dose–response relation between an individual's number of chronic conditions and risks of maternal mortality, severe maternal morbidity (SMM) and postpartum hospitalisation [10, 11, 12, 13]. However, studies have largely relied on birth hospitalisation records [10, 11, 12], wherein there may be under‐reporting of comorbidities due to the attention of the healthcare encounter on the birth [14]. Research is needed using data that allow better ascertainment of pre‐pregnancy MCC via population‐based linkage of health records over time. Prior studies have also analysed outcomes in relation to a simple count of maternal chronic conditions [10, 11, 12, 13, 14]; details are needed about whether the impact of MCC on maternal health outcomes differs by MCC complexity and how specific conditions cluster together. Outside of pregnancy, MCC impacts health outcomes more profoundly when it arises from disparate conditions (e.g., depression, diabetes and asthma), rather than closely related ones, likely due to the complexity of disease management across multiple specialists [15, 16]. Clustering of cardiometabolic conditions (i.e., cardiac disease, chronic hypertension, diabetes mellitus and obesity—which each individually carry high risks of mortality) also has a greater impact on risk of death than other MCC [17, 18]. Whether such patterns persist for maternal health outcomes is currently unknown.

In response to these knowledge gaps, we used longitudinally linked, population‐based data from Ontario, Canada, to assess risks of emergency department (ED) use, hospitalisation and SMM or maternal mortality (‘SMM‐M’) during pregnancy and the early postpartum period associated with 0, 1, 2 or ≥ 3 pre‐pregnancy chronic conditions. The outcomes were also evaluated by MCC complexity and the co‐occurrence of cardiometabolic chronic conditions.

## 2 Methods

### 2.1 Study Design and Data Sources

This population‐based cohort study used linked ICES and Better Outcomes Registry and Network (BORN) health administrative data for all of Ontario. Ontario is Canada's largest province, with 16 million residents and 140 000 births annually. Under a universal healthcare plan, Ontario residents have access to all essential healthcare, including general practitioner, specialist and emergency and hospital care related to chronic disease and pregnancy, at no direct cost. We accessed and analysed datasets at ICES containing complete and valid information on outpatient physician visits, ED use and hospital admissions, as well as basic sociodemographic data (Table S1) [19]. These datasets are linked using a unique encoded identifier, including to the BORN database for additional clinical birth data.

### 2.2 Study Cohort

The main cohort comprised females aged 13–54 years, who had a recognised pregnancy between April 1, 2012 and March 31, 2021, and, as residents of Ontario, were eligible for Ontario health insurance in the 2 years before the estimated date of conception. We included the full reproductive age range in our cohort, including adolescent pregnancies, in an effort to provide whole‐population estimations of risk, recognising the increasing burden of MCC in young people in addition to middle‐aged and older adults [2]. A recognised pregnancy comprised a livebirth or stillbirth at ≥ 20 weeks' gestation or a miscarriage or induced abortion at < 20 weeks, recorded in outpatient or inpatient healthcare settings (Table S2) [9, 20]. For births, the conception date was estimated by subtracting gestational age at delivery from the delivery date [9, 20]. (Gestational age is derived from a dating ultrasound for ~95% of births [21].) For a miscarriage or induced abortion, where dating information is missing, the estimated conception date was set using the median value from non‐missing data for that pregnancy type [20].

### 2.3 Study Measures

The primary approach for defining MCC was based on the number of chronic conditions recorded in health care encounters in the 2 years before the estimated date of conception. We applied a list of diagnoses used in prior studies focused on 16 chronic conditions based on their prevalence and healthcare costs [2, 22], and added 7 chronic conditions that may also influence pregnancy [23], for a total of 23 conditions measured using validated algorithms: [24, 25, 26, 27, 28, 29, 30, 31, 32, 33, 34, 35, 36, 37, 38, 39, 40, 41, 42, 43] alcohol and substance use disorders, asthma, cancer, cardiac arrhythmia, chronic hypertension, chronic kidney disease, chronic liver disease, chronic obstructive pulmonary disease, congestive heart failure, coronary artery syndrome, diabetes mellitus, HIV, inflammatory bowel disease, migraine, mood and anxiety disorders, multiple sclerosis, obesity, osteoarthritis, other mental illness, psychotic disorders, rheumatoid arthritis, stroke and systemic lupus erythematosus (Table S3). We enumerated the total number of conditions for each individual across a 2‐year lookback period and categorised each as having 0 (referent), 1, 2 or ≥ 3 chronic conditions.

Secondary study exposures were pre‐pregnancy complex MCC and the co‐occurrence of cardiometabolic chronic conditions. Complex MCC was defined as having ≥ 3 chronic conditions affecting ≥ 3 body systems, with body systems defined by the chapters of the International Classification of Diseases and Related Health Problems, version 10 [15, 16]. Individuals were then categorised as those with 0 chronic conditions, 1 chronic condition and MCC subdivided into non‐complex and complex MCC. Co‐occurring cardiometabolic chronic conditions of interest were cardiac arrhythmia, chronic hypertension, congestive heart failure, coronary artery syndrome, diabetes mellitus, obesity and stroke [18]. Individuals were categorised by the number of chronic conditions (0, 1, 2 or ≥ 3) of a cardiometabolic or non‐cardiometabolic nature.

The main outcomes were (a) maternal ED use, (b) maternal hospitalisation and (c) SMM‐M, the latter using an algorithm developed by the Canadian Perinatal Surveillance System [44] and adapted thereafter [45] (Table S4). ED use and hospitalisation were each assessed between the estimated date of conception and 42 days after the end of the pregnancy. For livebirths or stillbirths specifically, the ‘end’ of the pregnancy was set at the birth hospitalisation discharge, to account for varying lengths of stay and consistent assessment of post‐discharge ED use or readmission. SMM‐M was assessed from conception up to 42 days after the end of the pregnancy [45]. (A follow‐up of 42 days was chosen based on the standard World Health Organization definition of the ‘postpartum period’, which corresponds to when complications are most likely directly related to the pregnancy [46].) As secondary outcomes, ED use and hospitalisation were further categorised by (a) the ‘most responsible diagnosis’ at that encounter (i.e., obstetrical, medical or surgical, or psychiatric), and (b) the number of ED visits or hospitalizations in pregnancy or up to 42 days thereafter (i.e., 0, 1, 2 or ≥ 3).

Confounders were selected using a conceptual model informed by Misra's Integrated Perinatal Health Framework (Figure S1) [47]. Confounders included demographics (maternal age at conception, parity) and social environment characteristics (neighbourhood income quintile, rural residence defined by living in a region outside the commuting zone of an urban centre with a population of ≥ 10 000, immigrant or refugee status measured in the Immigration, Refugees and Citizenship Canada Permanent Residents database). We also measured pregnancy characteristics (pregnancy outcome, multi‐fetal status only for livebirths and stillbirths). We did not control for health behaviours such as smoking, alcohol use, diet and physical activity, as these were conceptualised as possible pathway variables [47].

### 2.4 Statistical Analyses

Baseline characteristics were described for those with 0, 1, 2 or ≥ 3 chronic conditions and compared using standardised differences [48]. Standardised differences with a value > 0.10 are considered important [48].

For the three main models, modified Poisson regression was used to calculate relative risks (RR) [49] for maternal ED use, maternal hospitalisation and SMM‐M in women with 0 (referent), 1, 2 or ≥ 3 conditions. Generalised estimating equations accounted for the potential clustering of more than one pregnancy per individual [50]. RRs were adjusted for demographic and social environment characteristics. For these main models, we also calculated the population attributable fraction (PAF), defined as the fraction of all cases with the outcome in the population that is attributable to the exposure, using a formula for polytomous exposures [51].

The aforementioned analyses were repeated for the exposures of (a) complex MCC and (b) the co‐occurrence of cardiometabolic chronic conditions.

In additional analyses, the main models were first restricted to those with a livebirth or stillbirth, in whom gestational age at birth was known. Second, we examined the outcomes as arising in pregnancy and separately in the postpartum period. Third, ED use and hospitalisation were evaluated by (a) the diagnostic nature of the encounter, using modified Poisson regression and (b) the number of encounters, using multinomial logistic regression.

Ascertainment of most chronic conditions included in our definition of MCC was based on the presence of diagnostic codes, and obesity was based on a combination of BMI data and diagnostic codes. Although most available algorithms have good sensitivity and specificity [24, 25, 26, 27, 28, 29, 30, 31, 32, 33, 34, 35, 36, 37, 38, 39, 40, 41, 42, 43], misclassification is possible. We therefore used the Rogan–Gladen equation [52] to conduct a quantitative bias analysis of potential misclassification of the conditions using known bias parameters from Ontario [24, 25, 26, 27, 28, 29, 30, 31, 32, 33, 34, 35, 36, 37, 38, 39, 40, 41, 42, 43] or estimated from similar algorithms in other regions [53].

We also calculated *E*‐values for the main models to assess the minimum magnitude of association an unmeasured confounder would need to have with the exposure and the outcome to bring the adjusted RRs to the null [54, 55].

Missingness was low (< 0.5% for demographics), so we did not use multiple imputation [56].

Analyses were performed using SAS version 9.4 (SAS Institute Inc., Cary, NC).

### 2.5 Ethics

ICES is a prescribed entity under Ontario's *Personal Health Information Protection Act* (PHIPA). Section 45 of PHIPA authorises ICES to collect personal health information, without consent, to inform the management of, evaluation of, allocation of resources to or planning the health system. The use of the data in this project is authorised under section 45 and approved by ICES' Privacy and Legal Office and the University of Toronto research ethics board (#44516).

### 2.6 Patient Involvement

This study used deidentified health administrative data collected by ICES. Identification or involvement of the patients whose data are included is not permitted.

## 3 Results

### 3.1 Study Cohort

During the study period, 894 042 women (65.1%) had no pre‐pregnancy chronic condition; 357 398 (26.0%) had 1; 94 427 (6.9%) had 2; and 27 326 (2.0%) had ≥ 3 chronic conditions before pregnancy (Table 1). In contrast to women with no chronic conditions, those with ≥ 3 chronic conditions were slightly younger, were more likely to live in low‐income neighbourhoods, and were less likely to be immigrants (Table 1).

**TABLE 1:** Characteristics of 1 373 193 women with 0, 1, 2 or ≥ 3 chronic conditions prior to a recognised pregnancy in Ontario, Canada, 2012–2021.

Characteristic	Number of chronic conditions (no. [% of all pregnancies])						
	0 conditions (*N* = 894 042 [65.1%])	1 condition (*N* = 357 398 [26.0%])	Standardised difference versus 0 conditions	2 conditions (*N* = 94 427 [6.9%])	Standardised difference versus 0 conditions	≥ 3 conditions (*N* = 27 326 [2.0%])	Standardised difference versus 0 conditions
Age, years, mean (SD)	30.34 ± 5.57	29.92 ± 5.87	0.07	29.59 ± 6.28	0.13	29.34 ± 6.70	0.16
13–24	135 932 (15.2)	66 529 (18.6)	0.09	20 986 (22.2)	0.18	6936 (25.4)	0.26
25–34	557 100 (62.3)	212 356 (59.4)	0.06	52 199 (55.3)	0.14	14 057 (51.4)	0.22
35–44	197 020 (22.0)	76 646 (21.4)	0.01	20 627 (21.8)	0.00	6056 (22.2)	0.00
45–54	3990 (0.4)	1867 (0.5)	0.01	615 (0.7)	0.03	277 (1.0)	0.07
Multiparous	490 969 (54.9)	203 981 (57.1)	0.04	54 307 (57.5)	0.05	15 733 (57.6)	0.05
Neighbourhood income quintile (Q)							
Q1 (lowest)	179 803 (20.1)	83 998 (23.5)	0.08	25 757 (27.3)	0.17	8895 (32.6)	0.29
Q2	177 955 (19.9)	73 916 (20.7)	0.02	20 509 (21.7)	0.04	5887 (21.5)	0.04
Q3	188 159 (21.0)	74 052 (20.7)	0.01	18 634 (19.7)	0.03	4970 (18.2)	0.07
Q4	189 922 (21.2)	69 921 (19.6)	0.04	16 754 (17.7)	0.09	4360 (16.0)	0.14
Q5 (highest)	155 903 (17.4)	54 400 (15.2)	0.06	12 445 (13.2)	0.12	3079 (11.3)	0.18
Missing	2300 (0.3)	1111 (0.3)	0.01	328 (0.3)	0.02	135 (0.5)	0.04
Rural residence	80 072 (9.0)	40 057 (11.2)	0.07	11 083 (11.7)	0.09	3242 (11.9)	0.10
Missing	1163 (0.1)	507 (0.1)	0.00	176 (0.2)	0.01	81 (0.3)	0.04
Immigrant status							
Long‐term resident	714 359 (79.9)	311 652 (87.2)	0.20	85 215 (90.2)	0.29	25 004 (91.5)	0.34
Family or economic class immigrant	155 306 (17.4)	37 299 (10.4)	0.20	7249 (7.7)	0.30	1783 (6.5)	0.34
Refugee	24 377 (2.7)	8447 (2.4)	0.02	1963 (2.1)	0.04	539 (2.0)	0.05
Pregnancy outcome							
Livebirth	658 875 (73.7)	277 902 (77.8)	0.09	73 481 (77.8)	0.10	20 879 (76.4)	0.06
Stillbirth	3284 (0.4)	1630 (0.5)	0.01	494 (0.5)	0.02	167 (0.6)	0.03
Miscarriage	119 136 (13.3)	41 111 (11.5)	0.06	10 771 (11.4)	0.06	3007 (11.0)	0.07
Induced abortion	112 747 (12.6)	36 755 (10.3)	0.07	9681 (10.3)	0.07	3273 (12.0)	0.02
Multi‐foetal pregnancy^a^	11 801 (1.8)	5549 (2.0)	0.01	1479 (2.0)	0.02	419 (2.0)	0.02

^a^ Measured only among livebirths and stillbirths.

### 3.2 Main Findings

Compared to those with no chronic conditions (rate 32.7%), the aRR for ED use rose proportionately higher with 1 (1.26, 95% CI 1.25–1.27), 2 (1.55, 95% CI 1.54–1.56), and ≥ 3 (1.86, 95% CI 1.85–1.88) chronic conditions (Table 2, upper). The PAFs indicated that 6.5%, 3.6% and 1.7% of cases of the outcome in the population could be attributed to having 1, 2 and ≥ 3 chronic conditions, respectively.

**TABLE 2:** (Main models). Risk of emergency department use, hospitalisation and severe maternal morbidity and mortality, from conception to 42 days postpartum, in women with 0, 1, 2 or ≥ 3 pre‐pregnancy chronic conditions.

Study outcome by number of chronic conditions	No. (%) with outcome	Unadjusted relative risk (95% CI)	Adjusted relative risk (95% CI)^a^
ED use			
0 chronic condition (*N* = 894 042)	291 931 (32.7)	1.00 (Ref.)	1.00 (Ref.)
1 chronic condition (*N* = 357 398)	153 926 (43.1)	1.28 (1.28–1.29)	1.26 (1.25–1.27)
2 chronic conditions (*N* = 94 427)	51 387 (54.4)	1.60 (1.58–1.61)	1.55 (1.54–1.56)
≥ 3 chronic conditions (*N* = 27 326)	18 314 (67.0)	1.94 (1.93–1.96)	1.86 (1.85–1.88)
Hospitalisation			
0 chronic condition (*N* = 894 042)	45 938 (5.1)	1.00 (Ref.)	1.00 (Ref.)
1 chronic condition (*N* = 357 398)	27 078 (7.6)	1.46 (1.44–1.48)	1.45 (1.43–1.47)
2 chronic conditions (*N* = 94 427)	10 343 (11.0)	2.09 (2.04–2.13)	2.06 (2.02–2.10)
≥ 3 chronic conditions (*N* = 27 326)	4716 (17.3)	3.25 (3.15–3.34)	3.18 (3.09–3.27)
Severe maternal morbidity or mortality (SMM‐M)			
0 chronic condition (*N* = 894 042)	13 903 (1.6)	1.00 (Ref.)	1.00 (Ref.)
1 chronic condition (*N* = 357 398)	7667 (2.1)	1.37 (1.34–1.41)	1.38 (1.35–1.42)
2 chronic conditions (*N* = 94 427)	2670 (2.8)	1.80 (1.73–1.88)	1.82 (1.75–1.90)
≥ 3 chronic conditions (*N* = 27 326)	1177 (4.3)	2.73 (2.58–2.90)	2.75 (2.59–2.92)

Abbreviation: CI, confidence interval.

^a^ Adjusted for maternal age (categorical), parity, neighbourhood income quintile, rural residence and immigrant/refugee status.

The same pattern was seen for hospital admissions in those with 1 (aRR 1.45, 95% CI 1.43–1.47), 2 (aRR 2.06, 95% CI 2.02–2.10) and ≥ 3 (aRR 3.18, 95% CI 3.09–3.27) chronic conditions, relative to no chronic condition (rate 5.1%) (Table 2, middle). Related PAFs were 9.6%, 6.0% and 3.6%.

For the outcome of SMM‐M, relative to those with 0 chronic conditions (rate 1.6%), the aRR was 1.38 (95% CI 1.35–1.42) with 1, 1.82 (95% CI 1.75–1.90) with 2 and 2.75 (95% CI 2.59–2.92) with ≥ 3 chronic conditions (Table 2, lower). The corresponding PAFs were 8.2%, 4.7% and 2.9%.

Compared to those without a chronic condition (rate 32.7%), the aRR for ED use was more pronounced for complex MCC (1.92, 95% CI 1.90–1.94) than non‐complex MCC (1.57, 95% CI 1.56–1.58) (Table 3, upper). The same was true for hospital admissions with complex MCC (aRR 3.16, 95% CI 3.05–3.27) in contrast to non‐complex MCC (aRR 2.17, 95% CI 2.13–2.22) (Table 3, middle), as well as for SMM‐M (aRR 2.92, 95% CI 2.72–3.14 vs. aRR 1.88, 95% CI 1.81–1.96) (Table 3, lower).

**TABLE 3:** Risk of emergency department use, hospitalisation and severe maternal morbidity and mortality, from conception to 42 days postpartum, in relation to pre‐pregnancy multiple chronic condition (MCC) complexity.

Study outcome by complexity of MCC	No. (%) with outcome	Unadjusted relative risk (95% CI)	Adjusted relative risk (95% CI)^b^
ED use			
0 chronic condition (*N* = 894 042)	291 931 (32.7)	1.00 (Ref.)	1.00 (Ref.)
1 chronic condition (*N* = 357 398)	153 926 (43.1)	1.28 (1.28–1.29)	1.26 (1.25–1.27)
Non‐complex MCC (*N* = 104 895)	58 186 (55.5)	1.62 (1.61–1.64)	1.57 (1.56–1.58)
Complex MCC (*N* = 16 858)^a^	11 515 (68.3)	1.97 (1.95–2.00)	1.92 (1.90–1.94)
Hospitalisation			
0 chronic condition (*N* = 894 042)	45 938 (5.1)	1.00 (Ref.)	1.00 (Ref.)
1 chronic condition (*N* = 357 398)	27 078 (7.6)	1.46 (1.44–1.48)	1.45 (1.43–1.47)
Non‐complex MCC (*N* = 104 895)	12 161 (11.6)	2.20 (2.16–2.25)	2.17 (2.13–2.22)
Complex MCC (*N* = 16 858)^a^	2898 (17.2)	3.23 (3.12–3.35)	3.16 (3.05–3.27)
Severe maternal morbidity or mortality (SMM‐M)			
0 chronic condition (*N* = 894 042)	13 903 (1.6)	1.00 (Ref.)	1.00 (Ref.)
1 chronic condition (*N* = 357 398)	7667 (2.1)	1.37 (1.34–1.41)	1.38 (1.35–1.42)
Non‐complex MCC (*N* = 104 895)	3066 (2.9)	1.86 (1.79–1.94)	1.88 (1.81–1.96)
Complex MCC (*N* = 16 858)^a^	781 (4.6)	2.94 (2.73–3.16)	2.92 (2.72–3.14)

Abbreviation: CI, confidence interval.

^a^ Complex MCC was defined as the co‐occurrence of ≥ 3 chronic conditions affecting ≥ 3 body systems, with systems defined by the chapters of the International Classification of Diseases and Related Health Problems version 10.

^b^ Adjusted for maternal age (categorical), parity, neighbourhood income quintile, rural residence and immigrant/refugee status.

While there was an increasing risk of ED use, hospitalisation and SMM‐M associated with the number of pre‐existing cardiometabolic and non‐cardiometabolic conditions, the most pronounced was seen with ≥ 3 cardiometabolic conditions (Table 4). For example, the aRR for SMM‐M was 5.45 (95% CI 4.29–6.91) with ≥ 3 cardiometabolic conditions, and 2.06 (95% CI 1.85–2.31) with ≥ 3 non‐cardiometabolic conditions (Table 4).

**TABLE 4:** Risk of emergency department use, hospitalisation and severe maternal morbidity and mortality, from conception to 42 days postpartum, in relation to the number of cardiometabolic and non‐cardiometabolic chronic conditions.

Study outcome by number of cardiometabolic and non‐cardiometabolic chronic conditions	No. (%) with outcome	Unadjusted relative risk (95% CI)	Adjusted relative risk (95% CI)^a^
ED use			
0 chronic condition (*N* = 894 042)	291 931 (32.7)	1.00 (Ref.)	1.00 (ref.)
1 non‐cardiometabolic condition (*N* = 223 237)	100 480 (45.0)	1.33 (1.32–1.34)	1.30 (1.30–1.31)
2 non‐cardiometabolic conditions (*N* = 45 567)	25 921 (56.9)	1.65 (1.64–1.67)	1.57 (1.56–1.58)
≥ 3 non‐cardiometabolic conditions (*N* = 10 657)	7398 (69.4)	1.99 (1.96–2.02)	1.85 (1.82–1.87)
1 cardiometabolic condition (*N* = 190 014)	84 829 (44.6)	1.33 (1.32–1.34)	1.31 (1.30–1.32)
2 cardiometabolic conditions (*N* = 9060)	4633 (51.1)	1.52 (1.49–1.55)	1.58 (1.55–1.62)
≥ 3 cardiometabolic conditions (*N* = 616)	366 (59.4)	1.76 (1.65–1.89)	1.91 (1.78–2.04)
Hospitalisation			
0 chronic condition (*N* = 894 042)	45 938 (5.1)	1.00 (Ref.)	1.00 (Ref.)
1 non‐cardiometabolic condition (*N* = 223 237)	16 826 (7.5)	1.44 (1.42–1.47)	1.44 (1.41–1.46)
2 non‐cardiometabolic conditions (*N* = 45 567)	5005 (11.0)	2.08 (2.02–2.14)	2.05 (1.99–2.11)
≥ 3 non‐cardiometabolic conditions (*N* = 10 657)	1860 (17.5)	3.24 (3.10–3.38)	3.18 (3.04–3.32)
1 cardiometabolic condition (*N* = 190 014)	16 937 (8.9)	1.72 (1.69–1.75)	1.69 (1.67–1.72)
2 cardiometabolic conditions (*N* = 9060)	1388 (15.3)	2.91 (2.77–3.06)	2.84 (2.70–2.99)
≥ 3 cardiometabolic conditions (*N* = 616)	121 (19.6)	3.73 (3.16–4.39)	3.59 (3.04–4.23)
Severe maternal morbidity or mortality (SMM‐M)			
0 chronic condition (*N* = 894 042)	13 903 (1.6)	1.00 (Ref.)	1.00 (Ref.)
1 non‐cardiometabolic condition (*N* = 223 237)	4120 (1.8)	1.18 (1.14–1.22)	1.20 (1.16–1.24)
2 non‐cardiometabolic conditions (*N* = 45 567)	1016 (2.2)	1.42 (1.34–1.52)	1.47 (1.38–1.56)
≥ 3 non‐cardiometabolic conditions (*N* = 10 657)	332 (3.1)	1.97 (1.76–2.20)	2.06 (1.85–2.31)
1 cardiometabolic condition (*N* = 190 014)	5474 (2.9)	1.84 (1.78–1.90)	1.83 (1.78–1.89)
2 cardiometabolic conditions (*N* = 9060)	510 (5.6)	3.56 (3.27–3.89)	3.25 (2.97–3.55)
≥ 3 cardiometabolic conditions (*N* = 616)	62 (10.1)	6.38 (5.02–8.11)	5.45 (4.29–6.91)

Abbreviation: CI, confidence interval.

^a^ Adjusted for maternal age (categorical), parity, neighbourhood income quintile, rural residence and immigrant/refugee status.

### 3.3 Additional Analyses

Findings were similar to the main models upon restricting the cohort to pregnancies ending in a livebirth or stillbirth (Table S5), and when outcomes were separately assessed within pregnancy and up to 42 days postpartum (Table S6).

For the outcomes of ED use and hospitalisations, the absolute rates of those encounters were more likely to be obstetrical, as well as medical or surgical, in nature, and less commonly for psychiatric reasons (Table S7). Even so, while the patterns for each rose with the number of antecedent chronic conditions, the aRRs were most pronounced for ED encounters of a psychiatric nature (13.79, 95% CI 13.03–14.60) and hospitalisations of a psychiatric nature (32.02, 95% CI 28.58–35.88) in women with ≥ 3 chronic conditions (Table S7).

Finally, in the multinomial logistic regression analysis, there was a higher odds of having multiple ED and hospitalisation encounters in relation to having a greater number of pre‐pregnancy chronic conditions (Table S8). Specifically, with ≥ 3 chronic conditions vs. none, the adjusted odds ratio of having ≥ 3 ED encounters was 10.15 (95% CI 9.75–10.56), and ≥ 3 hospitalisations was 15.85 (95% CI 13.95–18.00) (Table S8).

### 3.4 Quantitative Bias Analyses

The observed and bias‐adjusted prevalence estimates for each condition included in the definition of MCC were similar for some conditions and different for others, depending on the sensitivity/specificity of the algorithm. The median relative bias comparing observed and bias‐adjusted prevalence estimates was −101.1% (interquartile range: −110.4%, −21.1%) (Table S9).

E‐values for the main models ranged from 1.83 to 5.81 for the aRR point estimates and 1.81 to 5.63 for the lower bound of the 95% CI (Table S10).

## 4 Discussion

### 4.1 Main Findings

This population‐based study in Ontario, Canada found a dose–response relationship between the number of chronic conditions in the 2 years before pregnancy and the risk of ED use, hospitalisation and SMM‐M within and after pregnancy. Risks were most pronounced for complex MCC and cardiometabolic MCC. Notably, women with ≥ 3 chronic conditions had a 13 times higher risk of ED use for a psychiatric reason and a 32 times higher risk of hospitalisation for a psychiatric indication, compared to those with no chronic conditions.

### 4.2 Strengths and Limitations

Study strengths include the use of whole‐population data on all recognised pregnancies, with validated algorithms to identify most conditions [24, 25, 26, 27, 28, 29, 30, 31, 32, 33, 34, 35, 36, 37, 38, 39, 40, 41, 42, 43].

Our study was conducted in the context of a universal health care system; risks associated with MCC may be even greater in multi‐payer systems where financial barriers to care might lead to even greater risks of adverse outcomes.

In the derivation of our cohort, miscarriages occurring without a healthcare encounter were missed, as were births outside of hospital—2% of births in Ontario [57].

We used a similar definition of MCC as in prior studies [2, 9], which includes conditions with a high prevalence and related healthcare costs [22, 23]. We missed some rarer conditions (e.g., epilepsy) that could be relevant to pregnancy. However, studies have shown that even when using an exhaustive list of conditions, only a select number contribute to MCC [58]. We could not assess the severity of each condition. However, indices that weight conditions according to severity were mostly developed for older adults, and obstetric comorbidity indices largely include conditions arising in pregnancy (e.g., gestational hypertension) [59]. Moreover, studies have shown that simple counting of the number of chronic conditions, as in our study, has a similar performance to weighted indices in predicting death and hospitalisation [60]. It is possible that some individuals did not seek health care for their chronic condition in the 2 years before conception, in which case they would have been misclassified as being unexposed. However, requiring MCC to predate the pregnancy was important in ensuring that SMM events such as myocardial infarction, renal failure and stroke were not misclassified as MCC. On the other hand, we did not require a longer lookback (e.g., 5 years), as this would have required women to be residents of Ontario during this time, thereby disproportionately excluding immigrants.

MCC is associated with elevated risk of preterm birth [61]. It is therefore possible that women with MCC had less opportunity to experience an adverse maternal outcome than their peers due to shorter gestations. If so, the RRs observed herein are likely conservative.

Finally, while certain demographic and social environment variables were adjusted for, race/ethnicity, level of education and quality of care were not, raising the possibility of unmeasured confounding. However, estimation of *E*‐values [54, 55] indicated that unmeasured confounding would need to be very strong to explain the observed findings.

### 4.3 Interpretation

Few studies have examined the relationship between MCC and maternal health outcomes. In three studies using data from the US National Inpatient Sample [10, 11, 12], a dose–response relationship was seen between MCC and risk of SMM‐M, overall [10, 12] and after stratifying by race/ethnicity [11]. A fourth US study used data from a tertiary care centre and observed elevated risks of SMM‐M and hospital readmission in women with MCC versus those with 1 or 0 chronic conditions [13].

While the current findings echo those of the aforementioned research, several novel contributions were achieved. First, prior studies likely underestimated the risk of adverse maternal outcomes associated with MCC, as they were confined to live births and still births. Rather, women with MCC are more likely to have a miscarriage or induced abortion, which may herald greater ED and hospital use after pregnancy, or related SMM‐M [62]. Second, prior studies were limited to MCC and outcome data acquired within the birth hospitalisation [10, 11, 12], or from hospital admissions proximal to the delivery [13]. In our study, by capturing MCC data within a 2‐year lookback period and outcomes up to 42 days postpartum, including all outpatient, ED and inpatient encounters, MCC is better ascertained, and a more complete picture is provided of ED use, hospital admissions and SMM‐M across the perinatal period. Third, this study can enhance existing knowledge by providing nuanced data on the roles of MCC complexity and co‐occurring cardiometabolic conditions that each appear to confer a much greater risk for adverse maternal health outcomes and resource use.

Our findings have important implications for preconception and pregnancy‐related care. Preconception health promotion efforts should target known risk factors for MCC, including social determinants of health, poor nutrition and physical inactivity. In women with preexisting chronic conditions, including MCC, there should also be a focus on disease management (e.g., blood pressure, glycaemic control) to optimise their health going into pregnancy.

Like most healthcare [63], pregnancy‐related care is principally organised around the management of single acute conditions, rather than persons with MCC. Pregnant women with chronic conditions report fragmented care across obstetric, specialist and primary care providers [64]. Their obstetric providers likewise note that current organisational structures do not encourage joint deliberation or interprofessional collaboration [65, 66]. For example, patient care conferences that bring together obstetrics, anaesthesia, labour and delivery nursing and neonatology rarely occur, if at all, until mid‐pregnancy, and are reserved for the most complex cases in high‐risk centres. Our findings of elevated risks of ED use, hospitalisation and SMM‐M associated with MCC identify a group who stand to benefit from multidisciplinary, patient‐centred perinatal care. Importantly, their very high risk of psychiatric hospital use supports the inclusion of mental health resources within a multidisciplinary care framework. The occurrence of SMM‐M and multiple acute care encounters in those with MCC further highlights the necessity for better collaboration between obstetric and medical specialties, improved access to care when new or unexpected symptoms arise, and more frequent outpatient obstetric visits to better monitor these pregnancies. Recognising MCC in the preconception period or soon after conception may serve as an early activator of multidisciplinary planning within a formal pregnancy care plan [67]. Given the growing focus on multidisciplinary, patient‐centred care in other populations (e.g., frail older adults) [68], pregnancy represents a critical lost opportunity for more structured models of care for MCC.

## 5 Conclusion

While this study provides novel data on outcomes associated with any, complex and cardiometabolic MCC, consideration should be given to other combinations of conditions. MCC is socially patterned [2, 69], and healthcare costs and mortality rise disproportionately with an increasing number of chronic conditions among individuals of lower socioeconomic status [69]. Accordingly, future studies should determine which socioeconomic factors modify the relation between MCC and adverse maternal health outcomes. Finally, studies on MCC and pregnancy should focus on a broader array of perinatal outcomes, including infant outcomes [70], as well as the extended postpartum period [71] to better support new mothers with MCC and their infants.

## Author Contributions

H.K.B. and J.G.R. led the conception and design of the work; acquisition, analysis and interpretation of the data; and drafting of the work. K.F. contributed to the analysis and interpretation of the data and reviewed the work critically for important intellectual content. E.C., C.‐L.D., S.M.G., L.C.R., C.V., S.N.V. and W.P.W. contributed to the conception and design of the work, the interpretation of the data and reviewed the work critically for important intellectual content. All authors provided final approval of the version to be published and agree to be accountable for all aspects of the work in ensuring that questions related to the accuracy or integrity of any part of the work are appropriately investigated and resolved.

## Ethics Statement

This study was approved by the University of Toronto research ethics board (#44516).

## Conflicts of Interest

S.N.V. reports royalties from UpToDate Inc. for authorship of materials related to depression and pregnancy. E.C. reports paid membership on the Committee to Evaluate Drugs, which advises Ontario's Ministry of Health (MOH) on drug policy.

## Supporting information


**Table S1:** Description of ICES datasets.
**Table S2:** Ascertainment of recognised pregnancies.
**Table S3:** Ascertainment of multiple chronic conditions.
**Table S4:** Ascertainment of the study outcomes.
**Figure S1:** Study conceptual framework. (Black text = measurable in health administrative data; grey text = unmeasurable. Proximal determinants are conceptualised as mediators and are therefore not included in the multivariable models).
**Table S5:** Risk of emergency department use, hospitalisation and severe maternal morbidity and mortality, from conception to 42 days postpartum, in women with 0, 1, 2 or ≥ 3 pre‐pregnancy chronic conditions, restricted to a pregnancy ending in a livebirth or stillbirth.
**Table S6:** Risk of emergency department use, hospitalisation and severe maternal morbidity and mortality in women with 0, 1, 2 or ≥ 3 pre‐pregnancy chronic conditions, with outcomes separated by those arising in pregnancy and those arising within the 42‐day postpartum period.
**Table S7:** Risk of ED use or hospitalisation, from conception to 42 days postpartum, by the diagnostic nature of that encounter, among women with 0, 1, 2 or ≥ 3 pre‐pregnancy chronic conditions.
**Table S8:** Odds of having multiple ED or hospitalisation encounters, from conception to 42 days postpartum, among women with 0, 1, 2 or ≥ 3 pre‐pregnancy chronic conditions.
**Table S9:** Quantitative bias analysis for the prevalence of each chronic condition.
**Table S10:** *E*‐values for the potential impact of unmeasured confounding on the main models [54, 55].

## Data Availability

The dataset from this study is held securely in coded form at ICES. While legal data sharing agreements between ICES and data providers (e.g., healthcare organisations and government) prohibit ICES from making the dataset publicly available, access may be granted to those who meet pre‐specified criteria for confidential access, available at www.ices.on.ca/DAS (email: das@ices.on.ca). The full dataset creation plan and underlying analytic code are available from the authors upon request, understanding that the computer programs may rely upon coding templates or macros that are unique to ICES and are therefore either inaccessible or may require modification.
