# Supplementary material for: Multiple Chronic Conditions Before Pregnancy and Risk of Adverse Maternal Health Outcomes: Population‐Based Cohort Study
Source: BJOG. 2025 Sep 3;133(1):142–53. doi: 10.1111/1471-0528.18347 (PMC12676192; doi:10.1111/1471-0528.18347)
Supplement: Supplementary file 1 — Table S1: Description of ICES datasets. Table S2: Ascertainment of recognised pregnancies. Table S3: Ascertainment of multiple chronic conditions. Table S4: Ascertainment of the study outcomes. Figure S1: Study conceptual framework. (Black text = measurable in health administrative data; grey text = unmeasurable. Proximal determinants are conceptualised as mediators and are therefore not included in the multivariable models). Table S5: Risk of emergency department use, hospitalisation and severe maternal morbidity and mortality, from conception to 42 days postpartum, in women with 0, 1, 2 or ≥ 3 pre‐pregnancy chronic conditions, restricted to a pregnancy ending in a livebirth or stillbirth. Table S6: Risk of emergency department use, hospitalisation and severe maternal morbidity and mortality in women with 0, 1, 2 or ≥ 3 pre‐pregnancy chronic conditions, with outcomes separated by those arising in pregnancy and those arising within the 42‐day postpartum period. Table S7: Risk of ED use or hospitalisation, from conception to 42 days postpartum, by the diagnostic nature of that encounter, among women with 0, 1, 2 or ≥ 3 pre‐pregnancy chronic conditions. Table S8: Odds of having multiple ED or hospitalisation encounters, from conception to 42 days postpartum, among women with 0, 1, 2 or ≥ 3 pre‐pregnancy chronic conditions. Table S9: Quantitative bias analysis for the prevalence of each chronic condition. Table S10: E‐values for the potential impact of unmeasured confounding on the main models [54, 55]. [file BJO-133-142-s001.docx]

**TABLE OF CONTENTS**

**Table S1. Description of ICES datasets.**

**Table S2. Ascertainment of recognized pregnancies.**

**Table S3. Ascertainment of multiple chronic conditions.**

**Table S4. Ascertainment of the study outcomes.**

**Figure S1. Study conceptual framework.**

**Table S5. Risk of emergency department use, hospitalization, and severe maternal morbidity and mortality, from conception to 42 days postpartum, in women with** **0, 1, 2 or ≥ 3 pre-pregnancy chronic conditions, restricted to a pregnancy ending in a livebirth or stillbirth.**

**Table S6. Risk of emergency department use, hospitalization, and severe maternal morbidity and mortality in women with** **0, 1, 2 or ≥ 3 pre-pregnancy chronic conditions, with outcomes separated by those arising in pregnancy and those arising within the 42-day postpartum period.**

**Table S7. Risk of ED use or hospitalization, from conception to 42 days postpartum, by the diagnostic nature of that encounter, among women with** **0, 1, 2 or ≥ 3 pre-pregnancy chronic conditions.**

**Table S8. Odds of having multiple ED or hospitalization encounters, from conception to 42 days postpartum, among women with** **0, 1, 2 or ≥ 3 pre-pregnancy chronic conditions.**

**Table S9. Quantitative bias analysis for the prevalence of each chronic condition.**

**Table S10. E-values for the potential impact of unmeasured confounding on the main models.**

**Table S1. Description of ICES datasets.**

| **Dataset** | **Description** | **Dates** |
| --- | --- | --- |
| Better Outcomes Registry & Network (BORN) | Health behaviours in pregnancy and other clinical birth data | 2007-2021 |
| Canadian Institute for Health Information Discharge Abstract Database (CIHI-DAD) | Discharges from acute care hospitals, including the MOMBABY dataset, which identifies discharges for obstetrical deliveries ≥ 20 weeks gestation and links maternal and newborn data, coded using International Classification of Diseases and Related Health Problems (v. 10 since 2002) | 1988-present |
| Census database | Area-level profiles related to income and rurality | 1991-present |
| Immigration, Refugees and Citizenship Canada (IRCC) database | Immigration and refugee status | 1985-present |
| National Ambulatory Care Reporting System (NACRS) | Emergency department use, coded using International Classification of Diseases and Related Health Problems (v. 10 since 2002) | 2002-present |
| Ontario Drug Benefits (ODB) Database | Outpatient prescriptions for individuals receiving publicly funded drug benefits | 1990-present |
| Ontario Health Insurance Plan (OHIP) database | Claims submitted by physicians for reimbursement for ambulatory/hospital services provided to residents, coded using physician claims codes | 1991-present |
| Ontario Mental Health Reporting System (OMHRS) | Discharges from facilities with designated mental health beds, coded using Diagnostic and Statistical Manual of Mental Disorders codes | 2006-present |
| Ontario Registrar General Database (ORGD) | Vital statistics, including cause of death | 1990-present |
| Registered Persons Database (RPDB) | Dates of birth and death, sex, and postal code | 1990-present |
| Same-Day Surgery (SDS) database | Information about all day surgical procedures | 1991-present |

**Table S2. Ascertainment of recognized pregnancies.**

| **Pregnancy outcome** | **Codes** | **Data sources** |
| --- | --- | --- |
| Livebirth at 20+ weeks’ gestation | m_stillbirth=’F’ | MOMBABY |
| Stillbirth at 20+ weeks’ gestation | m_stillbirth=’T’ | MOMBABY |
| Induced abortion at < 20 weeks’ gestation* | 1. ICD-10: (O04 or O08) **if accompanied by** CCI: 5CA20, 5CA24, 5CA88, or 5CA89 [INATSTAT not equal to A] (include suspected diagnoses); 2. OHIP dx codes: 635; 3. OHIP feecodes: S752 or S785; 4. ODB DIN 2444038 | CIHI-DAD, CIHI-SDS, NACRS, OHIP |
| Miscarriage at < 20 weeks’ gestation* | 1. ICD-10: O00, O02.1, O03, or O20 (include suspected diagnoses; please do not include the DAD record if the KEY can be found in MOMBABY to avoid double-counting); 2. OHIP: (A921 **if accompanied by** DX 632, 633, or 634); 3. OHIP: dx codes 632, 633, 634, or 640; 4. OHIP: feecodes P001, A922, S756, S768, S770, or S784 | CIHI-DAD, CIHI-SDS, NACRS, OHIP |

Abbreviations: CCI = Canadian Classification of Health Interventions; CIHI-DAD = Canadian Institute for Health Information Discharge Abstract Database; ICD = International Classification of Diseases and Related Health Problems; ODB = Ontario Drug Benefits database; OHIP = Ontario Health Insurance Plan; SDS = Same-Day Surgery database

*Among records from DAD, SDS, and NACRS, if more than 1 record within 90 days, the first one is kept and the subsequent ones within 90 days are dropped to avoid misclassifying subsequent complications as a new pregnancy.

**Table S3. Ascertainment of multiple chronic conditions.**

| **Condition** | **Codes** | **Data source(s)** | **Body system** |
| --- | --- | --- | --- |
| Alcohol and substance use disorders | 2 physician visits: Psychiatrist [SPEC=19] and outpatient (LOCATION: O, L, H, P) and non-lab service [substr(FEECODE,1,1) ne ‘G’] OR Family physician / general practitioner / pediatrician [SPEC=00 or SPEC=26] and mental illness or addiction diagnosis code [DXCODE] and outpatient (LOCATION: O, L, H, P) and non-lab service [substr(FEECODE,1,1) ne ‘G’] OR pediatrician [SPEC=26] and undefined location (LOCATION = U) and MHA diagnostic code [DXCODE] and fee code (FEECODE=K122 or K123 or K704), where DXCODES = 291, 292, 303, 304; OR 1 hospital admission or ED visit: CIHI-DAD/NACRS: DX10CODE1 F10-F19, F55; OMHRS: Before 2016/17: AXIS1_DSM4CODE_DISCH1 = 291.x (all 291 codes, excluding 291.82), 292.x (all 292 codes, excluding 292.85), 303.x (all 303 codes), 304.x (all 304 codes), 305.x (all 305 codes), PROVDX_DSM4CODE_ADM1: 4; 2016/17-2018/19: DSM5CODE_DISCH1 = 291.x (all 291 codes), 292.x (all 292 codes), 303.x (all 303 codes), 304.x (all 304 codes), 305.x, Provisional = 16; 2019/20 to present: ICD10CMCODE_DISCH1=F10-F19, Z72.0; Provisional = 16^24^ | CIHI-DAD, NACRS, OHIP, OMHRS | Mental |
| Asthma | 2 physician visits (OHIP: 493) or 1 hospital admission (ICD-10: J45)^25^ | ASTHMA registry, from CIHI-DAD, OHIP | Respiratory |
| Cancer | 2 physician visits (OHIP: 140-239) or 1 hospital admission or ED visit (ICD-10: C00-C26, C30-C97)^26^ | Ontario Cancer Registry, from CIHI-DAD, OHIP | Neoplasms |
| Cardiac arrhythmia | 4 physician visits (OHIP: 427) (separated by 30 days) or 1 hospital admission or ED visit (ICD-10: I48.0, I48.1)^27^ | CIHI-DAD, NACRS, OHIP | Circulatory |
| Chronic hypertension | 1 hospital admission (ICD: I10-I13, I15) or 1 physician visit (OHIP: 401-405) followed by an additional physician visit or hospital admission (excluding gestational hypertension)^28^ | HYPER registry, from CIHI-DAD, NACRS, OHIP | Circulatory |
| Chronic kidney disease | 1 physician visit (OHIP: 403, 585) or hospital admission (ICD-10: E10.2, E11.2, E13.2, E14.2, I12, I13, N08, N18, N19)^29^ | CIHI-DAD, OHIP | Genitourinary |
| Chronic liver disease | 1 physician visit (OHIP: 571) or 1 hospital admission or ED visit (ICD-10: K70.0, K70.2, K70.3, K71.7, K73, K74.6, K75.4, K75.8, K75.9, K76.0, B18)^30^ | CIHI-DAD, NACRS, OHIP | Digestive |
| Chronic obstructive pulmonary disease | 1 physician visit (OHIP: 491, 492, 496) or 1 hospital admission (ICD-10: J41, J43, J44) and 35 years or older^31^ | Chronic Obstructive Pulmonary Disease registry, from CIHI-DAD, OHIP | Respiratory |
| Congestive heart failure | 1 physician visit (OHIP: 428 or feecode Q050) or 1 hospital admission (ICD-10: I50.0, I50.1, I50.9) followed by a 2^nd32^ | Congestive Heart Failure registry, from CIHI-DAD, OHIP | Circulatory |
| Coronary artery syndrome | 2 physician visits (OHIP: 411-414) or 1 hospital admission or ED visit (I20-I25)^33^ | CIHI-DAD, NACRS, OHIP | Circulatory |
| Diabetes | If < 19 years: 4 physician visits (OHIP: 250) or procedures (Q040, K029, K030, K045, K046) within 2 years and at least 1 of these before 19th birthday; if ≥ 19 years: 2 physician visits (OHIP: 250) or 1 procedure (Q040, K029, K030, K045, K046) or hospital admission (ICD-10: E10, E11, E13, E14) (excluding gestational diabetes)^34^ | Ontario Diabetes Dataset, from CIHI-DAD, OHIP | Endocrine and metabolic |
| HIV | 3 physician visits (OHIP: 042-044) or 1 hospital admission (ICD-10: B20-B24)^35^ | HIV registry, from CIHI-DAD, OHIP | Infectious |
| Inflammatory bowel disease | 2 years of OHIP eligibility and 5 physician visits (OHIP: 555, 556) or hospital admission or ED visits (ICD-10: K50, K51) OR < 2 years of OHIP eligibility and 3 physician visits (OHIP: 555, 556) or hospital admission or ED visits (ICD-10: K50, K51)^36^ | Ontario Crohn’s and Colitis Cohort dataset, from CIHI-DAD, NACRS, OHIP | Digestive |
| Migraine | 2 physician visits (OHIP: 346) or 1 hospital admission or ED visit (ICD-9ICD-10: G43, G44, G97.1, N95.1, R51)^37^ | CIHI-DAD, NACRS, OHIP | Nervous |
| Mood or anxiety disorders | 2 physician visits with a Psychiatrist [SPEC=19] and outpatient (LOCATION: O, L, H, P) and non-lab service [substr(FEECODE,1,1) ne ‘G’] OR Family physician / general practitioner / pediatrician [SPEC=00 or SPEC=26] and mental illness or addiction diagnosis code [DXCODE] and outpatient (LOCATION: O, L, H, P) and non-lab service [substr(FEECODE,1,1) ne ‘G’] OR pediatrician [SPEC=26] and undefined location (LOCATION = U) and MHA diagnostic code [DXCODE] and fee code (FEECODE=K122 or K123 or K704), where DXCODES = 296, 300, 309, 311; OR 1 hospital admission or ED visit: CIHI-DAD/NACRS: Before 2016/17: DX10CODE1 F30-F34, F38-F43, F48.8, F48.9, F53.0, F93.1-F93.2; 2016/17-present: DX10CODE1 F06.3, F06.4, F30-F34, F38-F43, F45.2, F53.0, F63.3, F93.0-F93.2, F94.0-F94.2; OMHRS: Before 2016/17: AXIS1_DSM4CODE_DISCH1 = 296.x (all 296 codes), 300, 300.0x, 300.2x, 300.3x, 300.4x, 301.13, 308.3x, 309.0x, 309.24, 309.28, 309.3x, 309.4x, 309.8x, 309.9x, 311; Provisional: 6, 7, 15. 2016/17-2018/19: DSM5CODE_DISCH1 = 293.83, 293.84, 296.x (all 296 codes), 300, 300.0x, 300.2x, 300.3x, 300.4x, 300.7x, 301.13, 308.3x, 309, 309.0x, 309.21, 309.24, 309.28, 309.3x, 309.4x, 309.81, 309.89, 309.9x, 311.x, 312.39, 313.23, 313.89, 625.4, 698.4x, Provisional = 3-7; 2019/20 to present: ICD10CMCODE_DISCH1=F06.3, F06.4, F06.8, F31-F34, F40.0-F40.2, F41.0, F41.1, F41.8, F41.9, F42.2-F42.4, F42.8, F42.9, F43.0-F43.2, F43.8, F43.9, F45.2, F63.3, F91.4, F94.2, F93.0, F94.0-F94.2; Provisional = 3-7^24^ | CIHI-DAD, NACRS, OHIP, OMHRS | Mental |
| Multiple sclerosis | 5 physician visits (OHIP: 340) or 1 hospital admission or ED visit (ICD-10: G35)^38^ | CIHI-DAD, NACRS, OHIP | Nervous |
| Obesity | If linked to BORN: BMI – NIDAY period = BMI: Overweight/obese ≥ 30.0, Other < 30.0; BMI – BIS period: MATERNAL_BMI = Overweight/obese ≥ 30.0; Other < 25.0. Where BMI/MATERNAL_BMI is missing: 2 physician visits (OHIP: 278) or 1 ED visit or hospital admission (ICD-10: E66)^9^ | BORN, CIHI-DAD, NACRS, OHIP | Endocrine and metabolic |
| Osteoarthritis | 2 physician visits (OHIP: 715) or 1 hospital admission or ED visit (ICD-10: M00-M03, M07, M10, M11-M25, M30-36, M65-M79)^40^ | CIHI-DAD, NACRS, OHIP | Musculoskeletal and connective tissue |
| Other mental illness | 2 physician visits: Psychiatrist [SPEC=19] and outpatient (LOCATION: O, L, H, P) and non-lab service [substr(FEECODE,1,1) ne ‘G’] OR Family physician / general practitioner / pediatrician [SPEC=00 or SPEC=26] and mental illness or addiction diagnosis code [DXCODE] and outpatient (LOCATION: O, L, H, P) and non-lab service [substr(FEECODE,1,1) ne ‘G’] OR pediatrician [SPEC=26] and undefined location (LOCATION = U) and MHA diagnostic code [DXCODE] and fee code (FEECODE=K122 or K123 or K704), where DXCODES = 301, 302, 306, 307, 309, 313-315; OR 1 hospital admission or ED visit: CIHI-DAD/NACRS: DX10CODE1 All other F06-F99 from MHAP algorithm not included in other categories, and excluding IDD diagnoses (F70-F73, F78, F79, F84.0, F84.1, F84.3-F84.9); OMHRS: All other OMHRS from MHAP algorithm not included in other categories, and excluding IDD diagnoses^24^ | CIHI-DAD, NACRS, OHIP, OMHRS | Mental |
| Psychotic mental illness | 2 physician visits: Psychiatrist [SPEC=19] and outpatient (LOCATION: O, L, H, P) and non-lab service [substr(FEECODE,1,1) ne ‘G’] OR Family physician / general practitioner / pediatrician [SPEC=00 or SPEC=26] and mental illness or addiction diagnosis code [DXCODE] and outpatient (LOCATION: O, L, H, P) and non-lab service [substr(FEECODE,1,1) ne ‘G’] OR pediatrician [SPEC=26] and undefined location (LOCATION = U) and MHA diagnostic code [DXCODE] and fee code (FEECODE=K122 or K123 or K704), where DXCODES = 295, 297, 298; OR 1 hospital admission or ED visit: CIHI-DAD/NACRS: DX10CODE1 F20 (excluding F20.4), F22-F25, F28-F29, F53.1; OMHRS: Before 2016/17: AXIS1_DSM4CODE_DISCH1 = 295.x (all 295 codes), 297.x (all 297 codes), 298.x (all 298 codes); Provisional = 5; 2016/17-2018/19: DSM5CODE_DISCH1 = 293.81, 293.82, 295.x (all 295 codes), 297.x (all 297 codes), 298.x (all 298 codes), Provisional = 2; 2019/20 to present: ICD10CMCODE_DISCH1=F20.81, F20.9, F22, F23, F25, F06.0-F06.2, F28, F29; Provisional = 2.^40^ | CIHI-DAD, NACRS, OHIP, OMHRS | Mental |
| Rheumatoid arthritis | 3 physician visits (OHIP: 714) with ≥ 1 by a specialist (rheumatologist, internal medicine specialist, orthopedic surgeon) or 1 hospital admission or ED visit (ICD-10: M05, M06)^41^ | Ontario Rheumatoid Arthritis Database, from CIHI-DAD, NACRS, OHIP | Musculoskeletal and connective tissue |
| Stroke | 2 physician visits (OHIP: 430-434, 436) or 1 hospital admission or ED visit (ICD-10: I60-I64)^42^ | CIHI-DAD, NACRS, OHIP | Circulatory |
| Systemic lupus erythematosus | 3 physician visits (OHIP: 710) with ≥ 1 by a specialist (rheumatologist) or 1 hospital admission (ICD-10: M32)^43^ | CIHI-DAD, OHIP | Musculoskeletal and connective tissue |

Abbreviations: CIHI-DAD = Canadian Institute for Health Information Discharge Abstract Database; DSM = Diagnostic and Statistical Manual of Mental Disorders; ICD = International Classification of Diseases and Related Health Problems; NACRS = National Ambulatory Care Reporting System; OHIP = Ontario Health Insurance Plan; OMHRS = Ontario Mental Health Reporting System

**Table S4. Ascertainment of the study outcomes.**

| **Outcome** | **Description** |
| --- | --- |
| Maternal emergency department (ED) use | Any ED visit occurring between the estimated date of conception up to ≤ 42 days after discharge from the delivery hospitalization, identified in NACRS. We excluded scheduled ED visits and individuals who left the ED without being seen, but included transferred ED visits. We included all ED visits, regardless of whether or not they were admitted to hospital from the ED (including for the birth hospital admission). |
| Maternal hospital admission | Any admission occurring between the estimated date of conception up to ≤ 42 days after discharge from the delivery hospitalization, identified in CIHI-DAD and OMHRS. To ensure we captured hospitalizations at the end of the postpartum period, continue following beyond 42 days postpartum to identify and classify hospitalizations; as long as the admission date fell within the 42-day period after delivery discharge, we counted it. We excluded the birth/miscarriage/induced abortion hospitalization but included transfers immediately after the delivery hospitalization (e.g., transfers from an obstetrics bed to psychiatry bed). |
| Severe maternal morbidity or mortality | A composite of 40 diagnostic (e.g., eclampsia) and procedural (e.g., assisted ventilation) indicators occurring between the estimated date of conception up to ≤ 42 days after the end date of the pregnancy, identified in CIHI-DAD |
| Acute renal  failure | Acute renal failure: O90.4, N17, N19, N99.0  Dialysis: 1.PZ.21^^ |
| Assisted  ventilation | Assisted ventilation through endotracheal tube: 1.GZ.31.CA-ND  Assisted ventilation through tracheostomy: 1.GZ.31.CR-ND [SMM26] |
| Cardiac  conditions | Cardiac arrest and resuscitation: I46, I49.0, 1.HZ.09, 1.HZ.30  Cardiac complications of anesthesia: O74.2, O89.1  Cardiomyopathy: O90.3, I42, I43  Myocardial infarction: I21, I22  Pulmonary edema and heart failure: I50, J81 |
| Cerebrovascular  accidents | Cerebral venous thrombosis in pregnancy, or in the puerperium: O22.5 or O87.3  Cerebrovascular diseases: subarachnoid and intracranial hemorrhage, cerebral infarction, stroke: I60-I64 |
| Embolism,  shock, DIC | Disseminated intravascular coagulation: D65  Obstetric embolism: O88  Obstetric shock: O75.1, R57, T80.5, T88.6 |
| Hysterectomy | Hysterectomy: 5.MD.60.RC, 5.MD.60.RD, 5.MD.60.KE, 5.MD.60.CB, 1.RM.89.LA, 1.RM.87.LA-GX; Note: 1.RM.89.LA is included only if codes 1.PL.74, 1.RS.74 or 1.RS.80 are NOT also present |
| Maternal  intensive care  unit admission | Use CIHI-DAD special care unit SCUadmdate1-6 variable |
| Sepsis | Puerperal sepsis: O85  Septicemia during labour: O75.3 |
| Severe  peripartum  hemorrhage | Antepartum hemorrhage with coagulation defect: O46.0  iCurettage with RBC transfusion: (5.PC.91.GA or 5.PC.91.GC or 5.PC.91.GD) + RBCTRNSF=‘Y’  Intrapartum hemorrhage with coagulation defect: O67.0  Intrapartum hemorrhage with RBC transfusion: O67 + RBCTRNSF=‘Y’  Placental abruption with coagulation defect: O45.0  Placenta previa with hemorrhage with RBC transfusion: O44.1 + RBCTRNSF=‘Y’  Postpartum hemorrhage with RBC transfusion, procedures to the uterus or hysterectomy: O72 + (RBCTRNSF=‘Y’ or [[1.RM.13^^ or 1.KT.51 or 5.PC.91.LA or 5.PC.91.HV} + RBCTRNSF=’Y’] or [5.MD.60.RC, 5.MD.60.RD, 5.MD.60.KE, 5.MD.60.CB, 1.RM.89.LA or [1.RM.87.LA-GX]); Note: 1.RM.89.LA is included only if codes 1.PL.74, 1.RS.74 or 1.RS.80 are NOT also present |
| Severe uterine  rupture | Rupture of the uterus with RBC transfusion, procedures to the uterus or hysterectomy: (O71.0 or O71.1) and (RBCTRNSF=‘Y’ or [1.RM.13^^ or 1.KT.51 or 5.PC.91.LA or 5.PC.91.HV] or [5.MD.60.RC, 5.MD.60.RD, 5.MD.60.KE, 5.MD.60.CB, 1.RM.89.LA] or [1.RM.87.LA-GX]); Note: 1.RM.89.LA is included only if codes 1.PL.74, 1.RS.74 or 1.RS.80 are NOT also present |
| Severe  preeclampsia,  eclampsia,  HELLP syndrome | Severe preeclampsia and HELLP syndrome: O14.1 or O14.2  Eclampsia: O15 |
| Surgical  complications | Complications of obstetric surgery and procedures: O75.4  Evacuation of incisional hematoma with RBC transfusion: 5.PC.73.JS + RBCTRNSF=‘Y’  Reclosure of caesarean wound with RBC transfusion: (5.PC.80.JM or 5.PC.80.JH) + RBCTRNSF=‘Y’  Repair of bladder, urethra, or intestine: 5.PC.80.JR, 1.NK.80^^, 1.NM.80^^ |
| Other | Acute abdomen: K35, K37, K65, N73.3, N73.5  Acute fatty liver with red blood cell (RBC) or plasma transfusion: O26.6 + RBCTRNSF=‘Y’ or PLSTRNSF=’Y’  Acute psychosis: F53.1, F23  Adult respiratory distress syndrome: J80  Cerebral edema or coma: G93.6, R40.2  Hepatic failure: K71-K72  Pulmonary, cardiac, and CNS complications of anaesthesia during pregnancy, the puerperium, or labour and delivery: O29.0, O29.1, O29.2, O89.0, O89.2, O74.0, O74.1, O74.3  Sickle cell anemia with crisis: D57.0  Status asthmaticus: J45.01, J45.11, J45.81, J45.91  Status epilepticus: G41  Surgical or manual correction of inverted uterus for vaginal births only: 5.PC.91.HP, 5.PC.91.HQ (exclude cesarean births, 5.MD.60) |
| Maternal  mortality | maternal death occurring between the estimated date of conception up to ≤ 42 days after the end date of the pregnancy |

Abbreviations: CIHI-DAD = Canadian Institute for Health Information Discharge Abstract Database; ICD = International Classification of Diseases and Related Health Problems; NACRS = National Ambulatory Care Reporting System; OMHRS = Ontario Mental Health Reporting System

**Figure S1. Study conceptual framework.** (Black text = measurable in health administrative data; grey text = unmeasurable. Proximal determinants are conceptualized as mediators and are therefore not included in the multivariable models.)

**
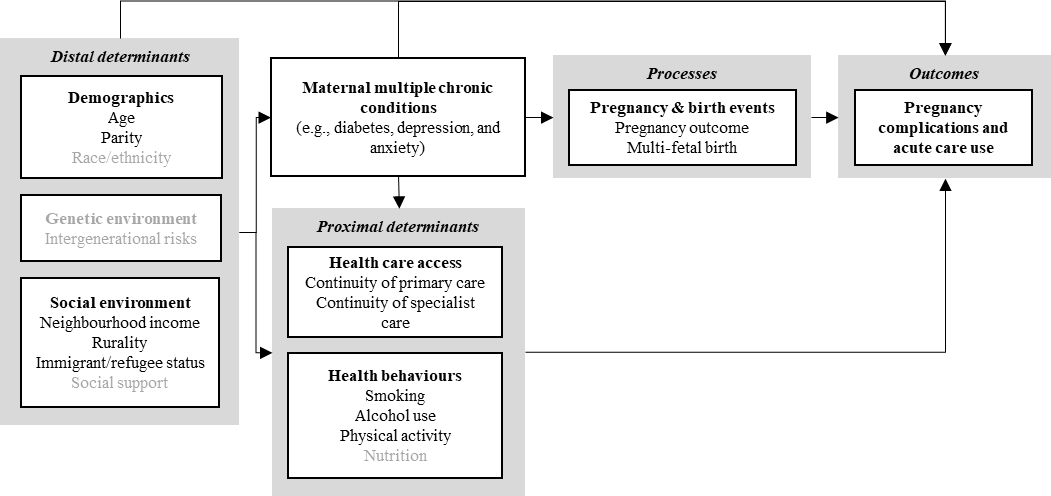
**

**Table S5. Risk of emergency department use, hospitalization, and severe maternal morbidity and mortality, from conception to 42 days postpartum, in women with** **0, 1, 2 or ≥ 3 pre-pregnancy chronic conditions, restricted to a pregnancy ending in a livebirth or stillbirth.**

| **Study outcome *by number of chronic conditions*** | **No. (%) with outcome** | **Unadjusted**  **relative risk**  **(95% CI)** | **Adjusted**  **relative risk**  **(95% CI)*** |
| --- | --- | --- | --- |
| **ED use** |  |  |  |
| *0 chronic condition (N = 662,159)* | 209,354 (31.6) | 1.00 (Ref.) | 1.00 (Ref.) |
| *1 chronic condition (N = 279,532)* | 120,380 (43.1) | 1.33 (1.32-1.34) | 1.29 (1.29-1.30) |
| *2 chronic conditions (N = 73,975)* | 40,943 (55.3) | 1.68 (1.67-1.69) | 1.60 (1.59-1.62) |
| *≥ 3 chronic conditions (N = 21,046)* | 14,398 (68.4) | 2.05 (2.03-2.08) | 1.93 (1.91-1.94) |
|  |  |  |  |
| **Hospitalization** |  |  |  |
| *0 chronic condition (N = 662,159)* | 38,540 (5.8) | 1.00 (Ref.) | 1.00 (Ref.) |
| *1 chronic condition (N = 279,532)* | 23,732 (8.5) | 1.44 (1.42-1.46) | 1.43 (1.40-1.45) |
| *2 chronic conditions (N = 73,975)* | 9,100 (12.3) | 2.06 (2.01-2.10) | 2.02 (1.97-2.06) |
| *≥ 3 chronic conditions (N = 21,046)* | 4,068 (19.3) | 3.18 (3.09-3.28) | 3.08 (2.99-3.18) |
|  |  |  |  |
| **Severe maternal morbidity or mortality** |  |  |  |
| *0 chronic condition (N = 662,159)* | 13,183 (2.0) | 1.00 (Ref.) | 1.00 (Ref.) |
| *1 chronic condition (N = 279,532)* | 7,298 (2.6) | 1.30 (1.27-1.34) | 1.31 (1.28-1.35) |
| *2 chronic conditions (N = 73,975)* | 2,557 (3.5) | 1.72 (1.65-1.79) | 1.73 (1.66-1.81) |
| *≥ 3 chronic conditions (N = 21,046)* | 1,071 (5.1) | 2.52 (2.36-2.68) | 2.53 (2.38-2.69) |

Abbreviations: CI = confidence interval.

*Adjusted for maternal age (categorical), parity, neighbourhood income quintile, rural residence, and immigrant/refugee status.

**Table S6. Risk of emergency department use, hospitalization, and severe maternal morbidity and mortality in women with** **0, 1, 2 or ≥ 3 pre-pregnancy chronic conditions, with outcomes separated by those arising in pregnancy and those arising within the 42-day postpartum period.**

| **Study outcome *by number of chronic conditions*** | **Outcome arising in pregnancy** | | | **Outcome arising postpartum** | | |
| --- | --- | --- | --- | --- | --- | --- |
|  | **No. (%) with outcome** | **Unadjusted relative risk**  **(95% CI)** | **Adjusted relative risk**  **(95% CI)*** | **No. (%) with outcome** | **Unadjusted relative risk**  **(95% CI)** | **Adjusted relative risk**  **(95% CI)*** |
| **ED use** |  |  |  |  |  |  |
| *0 chronic condition (N = 894,042)* | 242,792 (27.2) | 1.00 (Ref.) | 1.00 (Ref.) | 95,089 (10.6) | 1.00 (Ref.) | 1.00 (Ref.) |
| *1 chronic condition (N = 357,398)* | 131,657 (36.8) | 1.32 (1.31-1.32) | 1.29 (1.28-1.29) | 49,479 (13.8) | 1.28 (1.27-1.30) | 1.27 (1.25-1.28) |
| *2 chronic conditions (N = 94,427)* | 45,258 (47.9) | 1.68 (1.67-1.70) | 1.62 (1.61-1.63) | 17,344 (18.4) | 1.68 (1.66-1.71) | 1.64 (1.62-1.67) |
| *≥ 3 chronic conditions (N = 27,326)* | 16,648 (60.9) | 2.11 (2.09-2.14) | 2.01 (1.98-2.03) | 7,022 (25.7) | 2.33 (2.27-2.38) | 2.25 (2.20-2.30) |
|  |  |  |  |  |  |  |
| **Hospitalization** |  |  |  |  |  |  |
| *0 chronic condition (N = 894,042)* | 33,051 (3.7) | 1.00 (Ref.) | 1.00 (Ref.) | 13,716 (1.5) | 1.00 (Ref.) | 1.00 (Ref.) |
| *1 chronic condition (N = 357,398)* | 20,418 (5.7) | 1.53 (1.50-1.55) | 1.51 (1.48-1.54) | 7,383 (2.1) | 1.34 (1.30-1.38) | 1.35 (1.31-1.39) |
| *2 chronic conditions (N = 94,427)* | 8,013 (8.5) | 2.24 (2.19-2.30) | 2.20 (2.15-2.25) | 2,759 (2.9) | 1.89 (1.81-1.97) | 1.91 (1.83-1.99) |
| *≥ 3 chronic conditions (N = 27,326)* | 3,826 (14.0) | 3.65 (3.54-3.78) | 3.54 (3.43-3.66) | 1,210 (4.4) | 2.85 (2.69-3.02) | 2.87 (2.70-3.04) |
|  |  |  |  |  |  |  |
| **Severe maternal morbidity or mortality** |  |  |  |  |  |  |
| *0 chronic condition (N = 894,042)* | 10,955 (1.2) | 1.00 (Ref.) | 1.00 (Ref.) | 3,224 (0.4) | 1.00 (Ref.) | 1.00 (Ref.) |
| *1 chronic condition (N = 357,398)* | 6,063 (1.7) | 1.38 (1.34-1.42) | 1.39 (1.34-1.43) | 1,810 (0.5) | 1.40 (1.32-1.48) | 1.42 (1.34-1.51) |
| *2 chronic conditions (N = 94,427)* | 2,130 (2.3) | 1.83 (1.74-1.91) | 1.84 (1.75-1.93) | 631 (0.7) | 1.85 (1.70-2.01) | 1.89 (1.73-2.06) |
| *≥ 3 chronic conditions (N = 27,326)* | 947 (3.5) | 2.79 (2.61-2.98) | 2.79 (2.61-2.98) | 268 (1.0) | 2.70 (2.39-3.07) | 2.76 (2.43-3.13) |

Abbreviations: CI = confidence interval.

*Adjusted for maternal age (categorical), parity, neighbourhood income quintile, rural residence, and immigrant/refugee status.

**Table S7. Risk of ED use or hospitalization, from conception to 42 days postpartum, by the diagnostic nature of that encounter, among women with** **0, 1, 2 or ≥ 3 pre-pregnancy chronic conditions.**

| **Study outcome *by number of chronic conditions*** | **ED use** | | | **Hospitalization** | | |
| --- | --- | --- | --- | --- | --- | --- |
|  | **No. (%) with outcome** | **Unadjusted**  **relative risk**  **(95% CI)** | **Adjusted**  **relative risk**  **(95% CI)*** | **No. (%) with outcome** | **Unadjusted**  **relative risk**  **(95% CI)** | **Adjusted**  **relative risk**  **(95% CI)*** |
| **Encounter of an obstetrical nature** |  |  |  |  |  |  |
| *0 chronic condition (N = 894,042)* | 166,724 (18.6) | 1.00 (Ref.) | 1.00 (Ref.) | 38,787 (4.3) | 1.00 (Ref.) | 1.00 (Ref.) |
| *1 chronic condition (N = 357,398)* | 84,435 (23.6) | 1.24 (1.23-1.25) | 1.22 (1.22-1.23) | 22,085 (6.2) | 1.41 (1.39-1.43) | 1.41 (1.38-1.43) |
| *2 chronic conditions (N = 94,427)* | 28,436 (30.1) | 1.56 (1.54-1.58) | 1.52 (1.51-1.54) | 8,007 (8.5) | 1.92 (1.87-1.96) | 1.90 (1.86-1.95) |
| *≥ 3 chronic conditions (N = 27,326)* | 10,534 (38.5) | 1.98 (1.95-2.01) | 1.91 (1.88-1.94) | 3,378 (12.4) | 2.77 (2.68-2.86) | 2.73 (2.64-2.83) |
|  |  |  |  |  |  |  |
| **Encounter of a medical or surgical nature** |  |  |  |  |  |  |
| *0 chronic condition (N = 894,042)* | 181,954 (20.4) | 1.00 (Ref.) | 1.00 (Ref.) | 7,577 (0.8) | 1.00 (Ref.) | 1.00 (Ref.) |
| *1 chronic condition (N = 357,398)* | 107,008 (29.9) | 1.43 (1.42-1.44) | 1.38 (1.38-1.39) | 5,027 (1.4) | 1.65 (1.60-1.71) | 1.62 (1.56-1.68) |
| *2 chronic conditions (N = 94,427)* | 38,346 (40.6) | 1.90 (1.88-1.92) | 1.81 (1.79-1.82) | 2,072 (2.2) | 2.57 (2.45-2.70) | 2.49 (2.37-2.61) |
| *≥ 3 chronic conditions (N = 27,326)* | 14,719 (53.9) | 2.48 (2.45-2.51) | 2.32 (2.29-2.35) | 1,035 (3.8) | 4.41 (4.14-4.71) | 4.22 (3.95-4.51) |
|  |  |  |  |  |  |  |
| **Encounter of a psychiatric nature** |  |  |  |  |  |  |
| *0 chronic condition (N = 894,042)* | 3,585 (0.4) | 1.00 (Ref.) | 1.00 (Ref.) | 603 (0.1) | 1.00 (Ref.) | 1.00 (Ref.) |
| *1 chronic condition (N = 357,398)* | 4,692 (1.3) | 3.15 (3.01-3.29) | 2.89 (2.76-3.01) | 931 (0.3) | 3.80 (3.43-4.21) | 3.53 (3.19-3.91) |
| *2 chronic conditions (N = 94,427)* | 2,963 (3.1) | 7.39 (7.03-7.76) | 6.31 (6.00-6.63) | 814 (0.9) | 12.44 (11.19-13.83) | 10.82 (9.71-12.05) |
| *≥ 3 chronic conditions (N = 27,326)* | 2,011 (7.4) | 17.14 (16.20-18.13) | 13.79 (13.03-14.60) | 750 (2.7) | 39.17 (35.06-43.76) | 32.02 (28.58-35.88) |

Abbreviations: CI = confidence interval.

*Adjusted for maternal age (categorical), parity, neighbourhood income quintile, rural residence, and immigrant/refugee status.

**Table S8. Odds of having multiple ED or hospitalization encounters, from conception to 42 days postpartum, among women with** **0, 1, 2 or ≥ 3 pre-pregnancy chronic conditions.**

| **Study outcome *by number of chronic conditions*** | **ED encounter(s)** | | | **Hospitalization encounter(s)** | | |
| --- | --- | --- | --- | --- | --- | --- |
|  | **No. (%) with outcome** | **Unadjusted**  **odds ratio**  **(95% CI)** | **Adjusted**  **odds ratio**  **(95% CI)*** | **No. (%) with outcome** | **Unadjusted**  **odds ratio**  **(95% CI)** | **Adjusted**  **odds ratio**  **(95% CI)*** |
| **1 encounter** |  |  |  |  |  |  |
| *0 chronic condition (N = 559,896)* | 103,446 (18.5) | 1.00 (Ref.) | 1.00 (Ref.) | 25,295 (4.5) | 1.00 (Ref.) | 1.00 (Ref.) |
| *1 chronic condition N = 222,691)* | 48,206 (21.6) | 1.39 (1.37-1.41) | 1.36 (1.34-1.38) | 14,177 (6.4) | 1.45 (1.42-1.48) | 1.44 (1.41-1.47) |
| *2 chronic conditions (N = 59,683)* | 13,869 (23.2) | 1.86 (1.82-1.90) | 1.81 (1.77-1.85) | 5,311 (8.9) | 2.10 (2.04-2.17) | 2.08 (2.02-2.15) |
| *≥ 3 chronic conditions (N = 17,513)* | 3,760 (21.5) | 2.34 (2.24-2.44) | 2.27 (2.18-2.37) | 2,171 (12.4) | 3.15 (3.01-3.30) | 3.10 (2.96-3.25) |
|  |  |  |  |  |  |  |
| **2 encounters** |  |  |  |  |  |  |
| *0 chronic condition (N = 559,896)* | 44,779 (8.0) | 1.00 (Ref.) | 1.00 (Ref.) | 3,039 (0.5) | 1.00 (Ref.) | 1.00 (Ref.) |
| *1 chronic condition N = 222,691)* | 23,821 (10.7) | 1.58 (1.56-1.61) | 1.54 (1.51-1.56) | 2,077 (0.9) | 1.76 (1.67-1.86) | 1.75 (1.66-1.85) |
| *2 chronic conditions (N = 59,683)* | 7,828 (13.1) | 2.43 (2.36-2.49) | 2.33 (2.27-2.39) | 955 (1.6) | 3.15 (2.92-3.38) | 3.10 (2.88-3.34) |
| *≥ 3 chronic conditions (N = 17,513)* | 2,613 (14.9) | 3.75 (3.58-3.93) | 3.59 (3.42-3.76) | 531 (3.0) | 6.42 (5.85-7.05) | 6.26 (5.70-6.88) |
|  |  |  |  |  |  |  |
| **≥ 3 encounters** |  |  |  |  |  |  |
| *0 chronic condition (N = 559,896)* | 30,237 (5.4) | 1.00 (Ref.) | 1.00 (Ref.) | 783 (0.1) | 1.00 (Ref.) | 1.00 (Ref.) |
| *1 chronic condition N = 222,691)* | 22,582 (10.1) | 2.22 (2.18-2.27) | 2.09 (2.05-2.12) | 663 (0.3) | 2.18 (1.97-2.42) | 2.12 (1.91-2.35) |
| *2 chronic conditions (N = 59,683)* | 10,527 (17.6) | 4.84 (4.72-4.96) | 4.43 (4.31-4.54) | 394 (0.7) | 5.04 (4.46-5.69) | 4.77 (4.22-5.40) |
| *≥ 3 chronic conditions (N = 17,513)* | 5,209 (29.7) | 11.08 (10.66-11.52) | 10.15 (9.75-10.56) | 365 (2.1) | 17.13 (15.11-19.41) | 15.85 (13.95-18.00) |

Note: Multinomial logistic regression compares the odds of having 1, 2, or ≥ 3 encounters to 0 encounters, in relation to the number of pre-pregnancy chronic conditions. One pregnancy per individual was selected for this analysis to avoid any clustering effect.

Abbreviations: CI = confidence interval.

*Adjusted for maternal age (categorical), parity, neighbourhood income quintile, rural residence, and immigrant/refugee status.

**Table S9. Quantitative bias analysis for the prevalence of each chronic condition.**

| **Condition** | **P_pt_** | **Sensitivity** | **Specificity** | **P_adj_** | **RB** | **C_0_** | **C_α + 10%_** | **C_α - 5%_** |
| --- | --- | --- | --- | --- | --- | --- | --- | --- |
| Alcohol and substance use disorders | 4.0 | 61^a^ | 99^a^ | 1.5 | 26.7 | 2.5 | 2.2 | 2.7 |
| Asthma | 4.7 | 81^25^ | 81^25^ | 25.0 | -114.0 | 50.0 | 43.5 | 54.7 |
| Cancer | 1.2 | 87^a^ | 93^a^ | 7.8 | -110.3 | 35.0 | 25.7 | 44.3 |
| Cardiac arrhythmia | 0.2 | 81^27^ | 99^27^ | 1.6 | -81.5 | 5.0 | 3.7 | 6.3 |
| Chronic hypertension | 2.6 | 72^28^ | 95^28^ | 5.8 | -118.9 | 15.2 | 12.8 | 17.0 |
| Chronic kidney disease | 0.4 | 18^29^ | 98^29^ | 11.3 | -101.8 | 2.4 | 2.3 | 2.4 |
| Chronic liver disease | 0.2 | 95^a^ | 99^b^ | 0.9 | -123.5 | 16.7 | 6.9 | 95.0 |
| Chronic obstructive pulmonary disease | 1.4 | 57^a^ | 95^a^ | 9.4 | -101.1 | 10.4 | 9.5 | 11.0 |
| Congestive heart failure | 0.1 | 85^32^ | 97^32^ | 3.7 | -100.1 | 16.7 | 11.8 | 21.9 |
| Coronary artery syndrome | 0.3 | 85^a^ | 98^a^ | 2.3 | -104.4 | 11.8 | 8.1 | 15.8 |
| Diabetes mellitus | 3.1 | 90^34^ | 98^34^ | 1.0 | -207.6 | 16.7 | 10.0 | 27.1 |
| HIV | 0.1 | 96^35^ | 100^35^ | 0.1 | -4.0 | 0.0 | 0.0 | 0.0 |
| Inflammatory bowel disease | 0.5 | 77^36^ | 96^36^ | 4.8 | -110.4 | 14.8 | 11.9 | 17.3 |
| Migraine | 3.6 | 30^a^ | 99^37^ | 9.0 | -59.8 | 1.4 | 1.4 | 1.4 |
| Mood and anxiety disorders | 28.2 | 77^a^ | 99^a^ | 18.0 | -18.5 | 4.2 | 3.2 | 5.0 |
| Multiple sclerosis | 0.2 | 84^38^ | 100^38^ | 0.1 | -16.0 | 0.0 | 0.0 | 0.0 |
| Obesity | 15.8 | 74^a^ | 99^a^ | 16.0 | -20.3 | 3.7 | 3.0 | 4.3 |
| Osteoarthritis | 6.6 | 83^a^ | 95^a^ | 2.8 | -199.3 | 22.7 | 17.2 | 27.9 |
| Other mental illness | 1.1 | 77^a^ | 99^a^ | 1.3 | 52.0 | 4.2 | 3.2 | 5.0 |
| Psychotic mental illness | 0.5 | 92^40^ | 91^a^ | 10.6 | -101.9 | 52.9 | 36.7 | 71.3 |
| Rheumatoid arthritis | 0.2 | 78^41^ | 100^41^ | 0.3 | -22.0 | 0.0 | 0.0 | 0.0 |
| Stroke | 0.1 | 68^42^ | 98^42^ | 2.9 | -102.0 | 5.9 | 5.0 | 6.6 |
| Systemic lupus erythematosus | 0.1 | 68^43^ | 100^43^ | 0.1 | -32.0 | 0.0 | 0.0 | 0.0 |
|  |  |  |  |  |  |  |  |  |
| **Overall multimorbidity** | **8.9** | **73^b^** | **97^b^** | **8.4** | **5.6** | **10.0** | **8.3** | **11.4** |

^a^ Sensitivity and specificity values are based on a review of the literature of similar algorithms because validation studies from Ontario were not available or were based on significantly different underlying populations (e.g., the elderly).

^b^ The sensitivity and specificity values for multimorbidity as a whole are a weighted average of the sensitivities and specificities, respectively, of the individual chronic conditions.

Notes: P_pt_ = positive test proportion (i.e., the potentially misclassified prevalence estimate); P_adj_ = adjusted prevalence (i.e., bias-adjusted prevalence); RB = relative bias (i.e., the magnitude and direction of the possible misclassification bias; RB > 0 when P_pt_ > P_adj_ [positive bias] and RB < 0 when P_pt_ < P_adj_ [negative bias]); C_α_ = critical value of P_pt_ = P_adj_ at bias level α (presented for bias level 0%, 10%, and -5%)^52,53^

**Table S10. E-values for the potential impact of unmeasured confounding on the main models.^54,55^**

| **Study outcome *by number of chronic conditions*** | **Observed adjusted relative risk**  **(95% CI)*** | **E-value: point estimate** | **E-value: lower bound of the 95% CI** |
| --- | --- | --- | --- |
| **ED use** |  |  |  |
| *0 chronic condition (N = 894,042)* | 1.00 (Ref.) |  |  |
| *1 chronic condition (N = 357,398)* | 1.26 (1.25-1.27) | 1.83 | 1.81 |
| *2 chronic conditions (N = 94,427)* | 1.55 (1.54-1.56) | 2.47 | 2.45 |
| *≥ 3 chronic conditions (N = 27,326)* | 1.86 (1.85-1.88) | 3.12 | 3.10 |
|  |  |  |  |
| **Hospitalization** |  |  |  |
| *0 chronic condition (N = 894,042)* | 1.00 (Ref.) |  |  |
| *1 chronic condition (N = 357,398)* | 1.45 (1.43-1.47) | 2.26 | 2.21 |
| *2 chronic conditions (N = 94,427)* | 2.06 (2.02-2.10) | 3.54 | 3.46 |
| *≥ 3 chronic conditions (N = 27,326)* | 3.18 (3.09-3.27) | 5.81 | 5.63 |
|  |  |  |  |
| **Severe maternal morbidity or mortality** |  |  |  |
| *0 chronic condition (N = 894,042)* | 1.00 (Ref.) |  |  |
| *1 chronic condition (N = 357,398)* | 1.38 (1.35-1.42) | 2.10 | 2.04 |
| *2 chronic conditions (N = 94,427)* | 1.82 (1.75-1.90) | 3.04 | 2.90 |
| *≥ 3 chronic conditions (N = 27,326)* | 2.75 (2.59-2.92) | 4.94 | 4.62 |

Abbreviations: CI = confidence interval.

*Adjusted for maternal age (categorical), parity, neighbourhood income quintile, rural residence, and immigrant/refugee status.
